# Towards a more effective and efficient governance and regulation of nanomaterials

**DOI:** 10.1186/s12989-017-0235-z

**Published:** 2017-12-19

**Authors:** Tom Van Teunenbroek, James Baker, Aart Dijkzeul

**Affiliations:** 1 Ministry of Infrastructure and the Environment, Rijnstraat 8, 2515 XP, The Hague, The Netherlands; 2 Nanotechnology Industries Association (NIA), 143 Avenue de Terveuren, 1150 Brussels, Belgium; 3 Project Office ProSafe PB 73, Postbus 1, 3720 Bilthoven, BA Netherlands

## Abstract

The uncertainty regarding the effects and risks of nanomaterials on human health and the environment, and how they should be tested and assessed in the context of current regulations, is clearly holding back the full exploitation of the innovative potential of nanomaterials. To reduce this uncertainty, the European Union funded NANoREG and ProSafe projects (jointly referred to as N1P) have made a critical evaluation of methods to test and assess these risks in the context of the current registration, evaluation, authorisation and restriction of chemicals (REACH) regulation. Where essential methods were lacking, new ones have been developed. For several existing methods, adjustments have been proposed. Possible improvements to the REACH regulation have also been identified in these projects. The results of N1P have been translated into recommendations for (European) policy makers and regulators. Part of them have a “no regret” character, meaning that the proposed actions can be considered as necessary, feasible, effective and cost efficient. The recommended measures proposed for data quality and data management will create a more solid information basis for risk assessment of nanomaterials. When implemented, the recommendations regarding REACH will improve the application of REACH in both a legal and scientific sense. In practical terms however, the application of REACH will remain complex, time-consuming and costly. Besides that, adapting and specifying the information requirements and test methods in REACH for nanomaterials that are now on the market, will not solve the regulatory hurdles for next generation (nano) materials. To better align the dynamic character of developing new materials and the static character of regulations, it is recommended to explore possibilities of a more future proof approach for securing the safety of new (nano) materials.

## Introduction

Over the past 15 years there has been a significant global investment in nanosafety research. It has led to a better understanding of the effects of nanomaterials and the mechanisms which cause them. However, it still is difficult, if not impossible to come to unambiguous conclusions regarding the risks of most of the nanomaterials and nano-enabled products in the context of the current regulations.

**Fig. 1:**
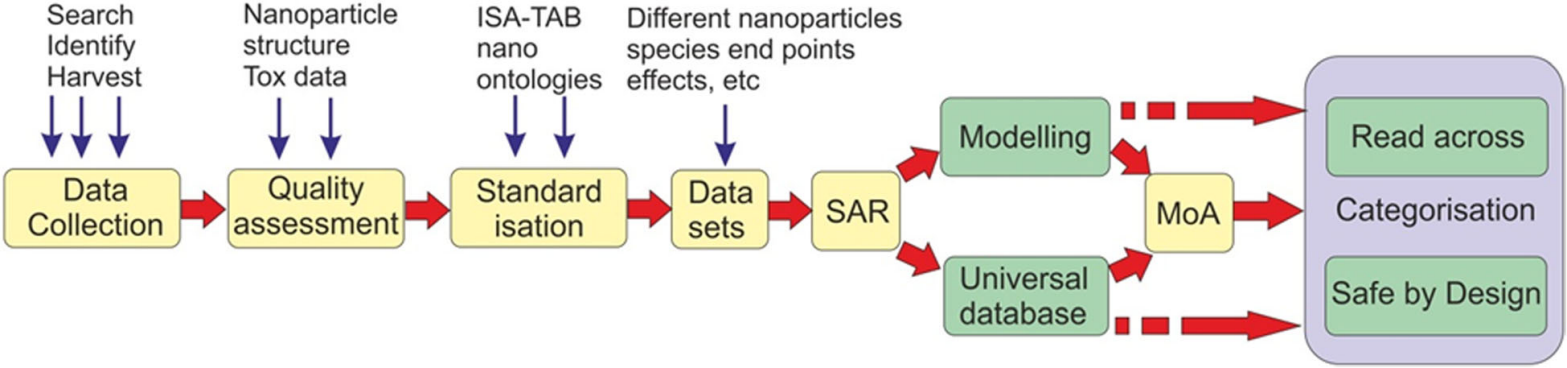
Example to show the general data collection framework for (Q) SAR studies, together with the issues that directly affect the reliability and suitability of the data collected for modelling purposes [1]

The main reason for this rather disappointing conclusion is that the research was predominantly “science-oriented” and not “regulatory-oriented”. Science-oriented research often results in experimental data that cannot be used in a regulatory context where data have to be well defined, standardised, reliable, reproducible and exchangeable. In this context it is important to note that not all traditional, standardised test methods are fit or relevant for nanomaterials. Additionally, methods specifically designed or adapted for testing nanomaterials are sometimes missing or not yet fully evaluated and harmonised.

A complicating factor why research in the past has not led to unambiguous results is the fact that the potential risks of nanomaterials may vary strongly during their life cycle, as a result of the nanomaterial undergoing major transformations. The hazard potential of pristine nanomaterials created during the production process differs from the effects of e.g. nanomaterials embedded in a coating or nanomaterials absorbed onto organic material in an aquatic environment. The presence of (sometimes high levels of) unintentionally produced nanomaterials (caused for example by abrasion or combustion processes) and naturally occurring nanomaterials (caused for example by volcanoes, rock weathering, etc.) further complicates both the distinction and judgement of exposure-related effects linked exclusively to manufactured nanomaterials. On top of this, a variety of analytical problems linked to aggregation, agglomeration and precipitation of nanomaterials in test media can lead to erratic or non-reproducible results.

Equally important, experience shows that the current legislation is not sufficiently robust to assess the risks of nanomaterials in an efficient and effective way. The absence of a robust, legal definition of nanomaterials in REACH leads to disputes as to whether specific information should be provided or not. The entry point for the REACH legislation (chemical identity) currently does not sufficiently cover the specific character of nanomaterials for which size, shape, coating and functionality are dominant factors determining hazard properties. Issues mentioned before like the absence of standardized test methods, and differences in hazard potential during the life cycle of nanomaterials lead to the current situation where it is impossible to come to unambiguous conclusions regarding the risk of most of the nanomaterials in a regulatory context.

N1P has developed recommendations for policy makers and regulators to solve or work around the problems and limitations mentioned above. This led to a White Paper entitled “To a more effective and efficient governance and regulation of nanomaterials” [1] for which the recommendations are summarized in this paper.

### Improving data quality and data management

The availability of harmonised and validated test methods for nanomaterials is a condition sine qua non for risk assessment in a regulatory context. They are needed to generate data that are reliable and comparable, and thus can be used and re-used for risk assessment and modelling the effects of nanomaterials. The NANoREG project [2] has developed a great number of test methods varying in status from proof of concept to validated, and that now can be made operational for use in a regulatory context (Fig. 1).

- In order to establish correlations between nanomaterial properties and the key interactions or endpoints in humans and the environment, sufficient well-defined experimental data on environmental, health and safety effects of nanomaterials (nanoEHS) are needed. Such data can serve as the basis for Quantitative Structure-Activity Relationships (QSARs), models, Adverse Outcome Pathways (AOPs) and read-across. These methods and tools can make risk assessment less time consuming and less costly, and are essential for implementing Safe by Design practises. Since reliable test methods have now been developed, it is time to generate these experimental data.
- There is a need for demand-driven projects to generate experimental nanoEHS data. Such projects should include adequately characterized materials that have different properties and include appropriate assays for examining interactions or endpoints. Materials that should be included in such a project are (a) “real-world” materials, (b) well-characterized reference materials of varied size, shape, aspect ratio, surface charge, and surface functionality and (c) standard materials for calibrating various assays and measurement tools.

Apart from the need to improve the quality and comparability of experimental nanoEHS data, the possibilities to use nanoEHS data outside and beyond a project need to be improved. This includes an agreement on opening up data, standards for data logging, ontology for nanosafety research and a database. This system can be expanded to a data management arrangement that should be used by the whole nanosafety community.

- Funding bodies should introduce and enforce an obligation to share the results of nanosafety research as a condition for funding project partners. The obligation should include uploading experimental nanoEHS data in a standardised (ISA-TAB-Nano logic, [3, 4]) way. A valid exemption to this rule would be nanosafety information generated for or by industry with a clearly competitive character.
- There is a need for allocating resources for the development and maintenance of a sustainable system for advanced nanoEHS data management, including providing or organising structural funding. Such an advanced system should include the further development and management of ontology, data entry provisions, facilities for storing and querying data, and providing a check on data quality (data curation). This last should be aimed at avoiding or repairing reporting errors as well as judging the regulatory appropriateness of experimental data.

Many of the finalised and current nanosafety projects are the result of national or international “Calls” that defined their goals in rather general terms. They often are not specific with respect to research topics that are relevant for using the results in a regulatory context, such as materials to be used, test methods to be applied and data logging. This limits the use of the projects’ results and experimental data for regulatory purposes like grouping and read-across, and for the development of QSARs. A more “top-down” approach, by precisely defining a call with respect to the basic conditions for proposals, or by tendering a well-defined project, would increase the impact of the results of nanosafety projects. Not only a top-down approach can help in a better alignment to regulatory needs, but more interaction with/involvement of regulatory bodies can also facilitate here. These regulatory bodies also need to clearly indicate where they need scientific knowledge or input.

- Where possible, calls for nanosafety projects should be far more specific in giving clear instructions to ensure that data and results generated are of a type and form which allows their use in topics of regulatory relevance, such as choice of materials, test methods to be applied, SOPs and data management.

### Harmonised occupational exposure limits

Bearing in mind that many nano-innovators are small and medium enterprises (SMEs), as are their downstream users, the most effective and efficient way to manage occupational exposure is by establishing Occupational Exposure Limits (OELs). Several EU Member States are developing or have developed OELs.

A joint project in setting occupational exposure levels for which standardised methods exist (see Joint Document, [5]), with guidelines for studies to be employed for conducting risk assessment [6] determinations as well as for setting occupational exposure levels is strongly recommended.

- The European Commission (DG-EMPL) should initiate a concerted EU - MS effort in setting occupational exposure levels (OELs) for which standardised methods on how to derive these OELs exist. This should include guidelines for studies to be employed, both for conducting risk assessment determinations as well as for setting OELs.

### A realistic REACH for nanomaterials

The present dispute between ECHA and groups of REACH registrants with respect to the information requirements for nanomaterials illustrates that there is a need to create a more solid and unambiguous legal basis for requirements that are specific for nanomaterials. The White Paper [1] recommended modifications partially overlapping with already ongoing adaptations of REACH Annexes and guidance documents proposed by the EC and ECHA.

- The European Commission and Member States should include a legal definition of nanomaterials in REACH, and should provide a more robust legal basis for additional nano-specific requirements. REACH Annexes and guidance documents should give clarity on the method(s) that can be applied for determining whether a material meets this definition.

To make REACH more applicable to nanomaterials from a scientific point of view, and to support possibilities for read-across and grouping, the information requirements in the Annexes and related guidance documents should be updated. Grouping and read-across of hazard and potential exposure data for nanomaterials should be further facilitated in order to increase the efficiency of risk assessment.

- The schemes for substance identification and substance specific profiles, irrespective of whether it is a manufactured nanomaterial or not, should be modified as suggested in NANoREG Deliverable 2.12. The morphological categorisation should be modified and aligned with the already developed (International Organization for Standardization) schemes and the OECD Guidance on grouping. Information on particle size distribution, shape, porosity, and surface chemistry should be added to the information requirements.

### Innovation in risk assessment

There is a great discrepancy between the time to market of new (applications of) nanomaterials and the time currently needed for risk assessment to comply with REACH (such as chronic exposure studies). Additionally, the costs of such tests are also high. Both constraints can be partially reduced by creating a more fundamental understanding of the mechanisms causing adverse effects (Mode of Action (MoA); AOPs). This makes it possible to predict the effect of nanomaterials on the basis of a more limited set of toxicity data.

Development of cheaper and faster test methods like in vitro High Throughput Screening (HTS) and in silico methods can also contribute to reducing the lead time for risk assessment and to reducing costs. It also contributes to the long-term aim to reduce animal testing.

- It is recommended initiating a project aimed at determining the MoA and AOPs for a number of nanomaterials that are representative for specific groups of nanomaterials while at the same time reducing the need for animal testing. Such an initiative could benefit from the experience of the SEURAT-1 Project [7] for nanomaterials in cosmetics that was also aimed at achieving a better understanding of mechanisms causing potential adverse effects, while developing methods to reduce animal testing.

### Exploring a more future proof approach

Implementation of the recommended measures outlined above with regard to data quality and data management will create a more solid “information basis” for risk assessment of nanomaterials. The recommendations regarding REACH will improve the application of this regulation in both a legal and scientific sense. In practice, the application of the regulation will remain complex, time-consuming and costly. Nevertheless adapting and specifying the information requirements and test methods in REACH for nanomaterials that are now on the market, will not solve the regulatory hurdles for the next generation of (nano) materials. In the actual regulatory context, every new generation material will necessitate adjusting test methods, information requirements and legislation. A process that, as the nanomaterial file illustrates, takes at least 10 years.

- To better align the dynamic character of developing new materials and the static character of regulations, the possibilities of a more future proof approach for securing the safety of new (nano)materials must be explored. Some possible options are mentioned in this N1P White Paper [1] and to some extent have been developed in the NANoREG Framework, such as the “nano-specific risk assessment approach” [8] and the “safe-by-design approach” [9, 10]. The first option is aligned with a more “concern-based testing approach” based on risk potentials that serve as indicators for potential hazards. It may include the application of functional assays as promising techniques for determining the potential hazard. The safe-by-design approach looks at ways to identify, and thus avoid, possible adverse effects of nanomaterials from the earliest stages of the innovation process onwards.
